# Modification of storage proteins in the barley grain increases endosperm zinc and iron under both normal and elevated atmospheric CO_2_

**DOI:** 10.1111/ppl.13624

**Published:** 2022-02-02

**Authors:** Yajie Gao, Daniel P. Persson, Eva Vincze, Jan K. Schjoerring

**Affiliations:** ^1^ Department of Plant and Environmental Sciences, Faculty of Science University of Copenhagen Frederiksberg Denmark; ^2^ Department of Agroecology, Faculty of Science and Technology, Research Centre Flakkebjerg Aarhus University Slagelse Denmark

## Abstract

Increasing atmospheric CO_2_ concentration is expected to enhance the grain yield of C_3_ cereal plants, while at the same time reducing the concentrations of minerals and proteins. This will lead to a lower nutritional quality and increase global problems associated with micronutrient malnutrition. Among the barley grain storage proteins, the C‐hordein fraction has the lowest abundance of sulfur (S) containing amino acids and is poorest in binding of zinc (Zn). In the present study, C‐hordein‐suppressed barley lines with reduced C‐hordein content, obtained by use of antisense or RNAi technology, were investigated under ambient and elevated atmospheric CO_2_ concentration. Grains of the C‐hordein‐suppressed lines showed 50% increase in the concentrations of Zn and iron (Fe) in the core endosperm relative to the wild‐type under both ambient and elevated atmospheric CO_2_. Element distribution images obtained using laser ablation‐inductively coupled plasma‐mass spectrometry confirmed the enrichment of Fe and Zn in the core endosperm of the lines with modified storage protein composition. We conclude that modification of grain storage proteins may improve the nutritional value of cereal grain with respect to Zn and Fe under both normal and future conditions of elevated atmospheric CO_2_.

## 1 INTRODUCTION

Cereals play a dominant role with respect to provision of dietary energy for humans and livestock. However, the grains of most cereal plants have nutritional shortcomings, including low levels of micronutrients and essential amino acids (Toride, 2002). Iron (Fe) and zinc (Zn) are essential micronutrients with a range of catalytic and structural functions (Anzellotti & Farrell, 2008; Caldelas & Weiss, 2017). In human beings, Zn and Fe deficiencies are estimated to threaten the health of two billion people worldwide, especially those who live on a diet based on cereals, in which the bioavailability of these two micronutrients is very low (Balk et al., 2019; Gibson, 2012; Stoltzfus & Dreyfuss, 1998; White & Broadley, 2011). This problem can to a large extent be attributed to the heterogeneous distribution of Fe and Zn in the cereal grain, where the concentrations of Zn and Fe are typically much lower in the core endosperm than in the embryo and aleurone layers (Cakmak, Kalayci, et al., 2010; Hansen et al., 2009; Lombi et al., 2011; Persson et al., 2016). The problem is exacerbated by the fact that the two grain fractions with the highest Zn and Fe concentration, i.e. the embryo and the aleurone, in most cases are removed during the milling process (Hansen et al., 2012; Xue et al., 2015). Increasing the concentration of Zn and Fe in the core endosperm of cereal species is therefore crucial to resolve the problems associated with micronutrient malnutrition. Barley (*Hordeum vulgare* L.) is one of the most important crops worldwide and is mostly used for animal feed and brewing of beer. Compared to common bread wheat (*Triticum aestivum* L.), which is hexaploid, barley has a smaller and less complex diploid genome, which makes it more suitable for genetic modifications aiming at exploring the mechanisms underlying micronutrient accumulation in the grain (Saisho & Takeda, 2011; Sreenivasulu et al., 2008).

The relatively low proportion of essential amino acids in cereal grains is related to the content of prolamins, which account for the major part of the total storage proteins. Prolamins are characterized by a high abundance of glutamine and proline, while being low in essential amino acids such as lysine and threonine. In barley, the prolamins are called hordeins and are further classified into four fractions, viz. the B and C‐hordeins accounting for 70%–80% and 10%–20% of the total grain hordeins, respectively, and the D and γ‐hordeins, which are minor components (Shewry & Tatham, 1990). With respect to S content, the B and γ‐hordeins are rich, the D‐hordeins are medium, and the C‐hordeins are poor, the latter primarily consisting of proline and glutamine with no or only a few cysteine/methionine residues (Lange et al., 2007; Shewry, 1995).

Issues regarding the nutritional quality of cereal grain have gained increasing attention (Cakmak & Kutman, 2018; Gregory et al., 2017; Postles et al., 2016). The problems of poor nutritional value may be intensified by increasing atmospheric carbon dioxide (CO_2_), which is predicted to reach 550 μmol mol^−1^ by the middle of this century and to surpass 700 μmol mol^−1^ by the end of the century (Long et al., 2004; Myers et al., 2014; Soares et al., 2019). In general, elevated atmospheric CO_2_ stimulates photosynthesis and productivity of C_3_ cereal plants, but negatively affects nitrogen (N) metabolism (Ainsworth & Long, 2005; Dier et al., 2018; Rubio‐Asensio & Bloom, 2017), leading to a decline in grain protein concentration (Bahrami et al., 2017; Tausz et al., 2017), and an altered composition of the grain proteins (Högy & Fangmeier, 2008; Ingvordsen et al., 2016; Panozzo et al., 2014; Uddling et al., 2018; Wroblewitz et al., 2014). Beside these changes, several cases of decreased mineral concentrations in milled grains induced by elevated atmospheric CO_2_ have been reported (Asif et al., 2017; Beleggia et al., 2018; Myers et al., 2014). A modeling study estimated that by 2050, a further 138 million people will be threaten by Zn deficiency due to the CO_2_‐induced reduction in Zn concentration of cereal grains (Myers et al., 2015). The grain quality of cereals must therefore be improved under future atmospheric CO_2_ conditions.

In order to improve the protein nutritional value of cereals, strategies using antisense constructs (Hansen et al., 2007; Lange et al., 2007; Schmidt et al., 2015, 2016) or double‐stranded RNA interference (RNAi) (Barro et al., 2016; Gil‐Humanes et al., 2008; Houmard et al., 2007; Sikdar et al., 2016) have been used to change the relative proportions of the major storage proteins. In barley, the antisense C‐hordein lines produced by Lange et al. (2007) had an average 20% reduction of C‐hordeins and a relatively high content of glutelin and B‐hordein fractions. These changes were accompanied by a decline in proline and glutamine as well as an increase in the content of lysine, threonine, and methionine. Using RNAi technology, up to 95% reduction of C‐hordein was achieved with a concomitant increase in B‐ and γ‐hordein (Sikdar et al., 2016). Grain storage proteins play a crucial role in binding Zn (Kutman et al., 2011; Persson et al., 2016; Regvar et al., 2011). Transcript analysis of barley hordeins indicated that the expression of genes encoding B‐hordeins was positively correlated with the Zn concentration, while the opposite was the case for genes encoding C‐hordeins (Uddin et al., 2014). In the endosperm, the B‐hordein fraction, and to a lesser extent the γ‐ and D‐hordeins have been found to be abundant in Zn, whereas C‐hordeins were not (Dionisio et al., 2018). Thus, modification of the proportions of Zn binding proteins in cereal grains may provide a potential for Zn biofortification. A positive correlation between grain protein, Zn, and Fe, has frequently been reported (Cakmak, Pfeiffer, et al., 2010; Persson et al., 2016; Raboy et al., 1984). Biofortification strategies embracing transporter engineering and/or conventional breeding have in several cases enabled increased Fe and Zn levels in the cereal grain (Beasley et al., 2019; Ludwig & Slamet‐Loedin, 2019; Menguer et al., 2017; Van der Straeten et al., 2020). However, it has not yet been investigated if the Zn and Fe concentration in the endosperm can be increased through engineering of the relative proportions of grain storage proteins.

The objective of the present study was to test the hypothesis that suppression of C‐hordein would lead to higher Zn and Fe concentrations in the barley grain. Barley lines with suppressed C‐hordein synthesis were cultivated in greenhouse experiments at ambient (400–500 ppm) and elevated atmospheric CO_2_ concentrations (about 800 ppm). The mature grains were analyzed for the concentrations and distribution of Zn and Fe as well as other mineral elements.

## 2 MATERIALS AND METHODS

### 2.1 Plant material and greenhouse experiment

Mature grains of wild‐type barley (*Hordeum vulgare* cv. Golden Promise), a C‐hordein‐RNAi line and a C‐hordein‐antisense line were used in the experiments. The two C‐hordein‐suppressed lines were generated by Agrobacterium mediated transformation of barley embryos with the expression vector containing 480 bp of the C‐hordein sequence (Gene Bank accession number: S66938). The RNAi line was generated by Sikdar et al. (2016), where details concerning the double‐stranded RNAi construct and the transformation procedure are specified. For more details concerning the antisense construct and the transformation procedure used to generate the C‐hordein antisense line, see Lange et al. (2007).

The grain element composition of the C‐hordein‐suppressed lines was studied in two independent greenhouse experiments, conducted from December 9, 2014 to April 19, 2015 (Experiment I) and from September 21, 2015 to February 14, 2016 (Experiment II). The T3 C‐hordein‐antisense line and the RNAi line were grown together with the wild‐type plants in two adjacent greenhouse cells with ambient (400–500 ppm) and elevated (700–800 ppm in Experiment I and 800–900 ppm in Experiment II) atmospheric CO_2_ concentrations, respectively. The greenhouse cells had an area of 50 m^2^, a height of 5 m, and were located at the experimental farm of University of Copenhagen, Taastrup, Denmark (55 N40′6.61″; 12E18′25.62″). The CO_2_ enrichment was maintained 24 h per day throughout the entire experimental period. Experiment I and II included 10 and 6 replicate plants, respectively. Seeds were germinated on filter paper, and one seed of each genotype was subsequently transferred to 2 L pots with soil containing 0.12 g inorganic N L^−1^ soil, pH 5.6–6.4 (Pindstrup 2, Ryomgaard, DK). In Experiment I, 0.06 g inorganic N L^−1^ soil was added to each pot to obtain inorganic N levels of 0.18 g L^−1^ soil, while in Experiment II, 0.08 g inorganic N L^−1^ soil was added to each pot, providing an inorganic N level of 0.2 g L^−1^ soil. N was added as NH_4_NO_3_ dissolved in 100 ml water solution, which was split in two equal dosages and applied at 28 days after germination (DAG) and 56 DAG. Plants were grown at a 20/16°C day/night (16/8 h) temperature regime with 70% relative air humidity and 400–450 and 450–500 μmol m^−2^ s^−1^ photosynthetic photon flux density in Experiment I and II, respectively, provided by natural light combined with LED lamps, the latter providing approximately 250 μmol m^−2^ s^−1^. All pots were given deionized water to maintain 70–80% of the soil water holding capacity every 4–5 days before stem elongation, every 2–3 days after stem elongation until the heading stage and thereafter less frequently during the maturation stage. All pots in each greenhouse cell were positioned randomly and rotated at least once per week. All heads of the plant in each pot, representing the individual replicate of each genotype, were harvested at maturity. All the grains were separated from the husk by hand, mixed, and subsequently dried and weighed for further analysis.

To separate the bran and the endosperm, a polishing process was performed by high speed shaking in a ball mill (RetschMM301). First, the embryo was gently loosened and removed by use of the tip of a scalpel. The grains were thereafter polished by shaking with quartz sand in 20 ml tubes, each containing 10 grains and 2000 mg of acid‐washed quartz sand. The tubes were mounted in racks, which were agitated at 30 Hz until the fused testa and aleurone tissue layers were polished off. Control tubes filled only with sand were included. About 25% of the dry matter from the whole grains was polished off. The remaining part of the grain (pure endosperm) was separated from the abraded material (the fused testa and aleurone tissue layers), weighed and collected for further analysis.

### 2.2 Analysis of protein concentrations

Hordeins were extracted from 50 mg of pulverized whole grain samples using 500 μL of extraction buffer (55% 2‐propanol, 1% glacial acetic acid, and 2% mercaptoethanol). The individual hordein fractions, i.e. C‐hordein, B/γ‐hordein, and D‐hordeins, were separated by SDS‐PAGE (Figure S1) and the percentage of band volume in each lane was quantified by image analysis, as previously described by Uddin et al. (2014) and Sikdar et al. (2016).

The total N concentration in dried, pulverized grain samples and in solutions with extracted hordeins was analyzed by Dumas combustion (Vario Macro elemental analyzer, Elementar Analysensysteme GmbH, Hanau, Germany). For the analysis of dried, pulverized grain samples (approximately 40 mg), acetanilide was used as reference material. The total grain protein concentration (% of grain DM) was calculated by multiplication of the N concentration with 5.4 (Mariotti et al., 2008). For the analysis of total N concentration in the solution with extracted hordeins, 50 μl of the extract was pipetted into a tin capsule and dried at room temperature, before being sealed for analysis. Acetanilide was used as reference material for quantification and instrument stabilization. Absolute values for the content of the different hordeins per unit grain weight and per unit grain protein were calculated on the basis of the N concentration of the extracts and the relative proportions of the different hordeins obtained in the image analysis of the SDS gels.

### 2.3 Analysis of total element concentrations in barley grains by ICP‐OES

The pure endosperm grain materials were digested using 2.5 mL of 70% HNO_3_ and 1 mL of 5% hydrogen peroxide (H_2_O_2_) per sample in an Ultrawave Microwave Acid Digestion System (Milestone UltraWAVE). Inductively coupled plasma optical emission spectrometry (ICP‐OES, Vista‐Pro Axial; Varian Pty Ltd.) was used to determine the Zn, Fe, Mn, Cu, Ca, Mg, K, P, and S concentrations in the digests. Milli‐Q water was used for all solutions, dilutions and wash procedures.

For total grain element concentrations, whole grains (4–5 g) were ground with zirconium balls by high‐speed shaking in a ball mill (RetschMM301), digested and analyzed following the same procedures as described for polished grains.

### 2.4 Laser ablation‐inductively coupled plasma‐mass spectrometry analysis of grains

Individual grains were put in the center of a rubber mold, and submerged in nonfrozen, but precooled optimal cutting temperature (OCT) medium (Sakura Finetek, Tokyo, Japan). The mold with its contents was immediately put in liquid N_2_ to freeze it. After freezing, the solid OCT block with a grain inside was transferred to a cryotome (Leica CM050S, St. Gallen, Switzerland; precooled to −30°C) for sectioning. Cross‐sections (20 μm thick) were cut with cryofilm (Cryofilm 2C, SectionLab), then transferred to and mounted onto glass slides (MembraneSlide 1.0 PEN, Carl Zeiss Microscopy).

An ArF excimer laser ablation unit (NWR193, New Wave Research, Fremont, CA) operating at 193 nm wavelength was used for the laser ablation‐inductively coupled plasma‐mass spectrometry (LA‐ICP‐MS) analyses. A Dual Concentric Injector (DCI, New Wave Research) was employed in order to improve the wash‐out time, i.e. the sample transfer from the ablation chamber to the ICP‐MS. The following settings were used: energy: 0.9–1.1 J cm^−2^ (30% of maximum energy), scan speed: 50 μm s^−1^, repetition rate: 40 Hz, and spot size 20 μm. The transfer gas (from LA unit to ICP‐MS) was helium (He), used with a flowrate of 750 ml min^−1^. All elemental signals were monitored and collected with an Agilent 7900 ICP‐MS (7900 ICP‐MS, Agilent technologies, Manchester, UK), operated in H_2_‐mode (2.5 ml min^−1^). Sample cone depth in the ICP‐MS was 4.0 mm and the ICP‐carrier gas (Ar) was set to 0.89 ml min^−1^. The isotopes analyzed were ^13^C, ^31^P, ^34^S, ^39^K, ^40^Ca, ^55^Mn, ^56^Fe, and ^66^Zn, using integration times of 0.08 (^13^C), 0.05 (^31^P, ^34^S), 0.04 (^40^Ca, ^55^Mn, ^56^Fe, and ^66^Zn), and 0.02 (^39^K) s. The scan cycle was 0.375 s. All data were normalized against endogenous carbon (^13^C) and processed with SigmaPlot version 13 (Systat Software Inc., London, UK). A spot size of 20 μm in diameter was enough to get sufficient signal strength of all elements that were included in the analysis.

### 2.5 Statistical analysis

The IBM SPSS Statistics software (version 22) was used to carry out the statistical analysis. The differences between genotypes within each treatment and the differences between ambient and elevated CO_2_ within each genotype were analyzed by two‐way analysis of variance (anova) with Fisher's lsd post hoc test. Differences were considered statistically significant at *P* <0.05.

## 3 RESULTS

### 3.1 Characterization of C‐hordein‐suppressed lines

**FIGURE 1:**
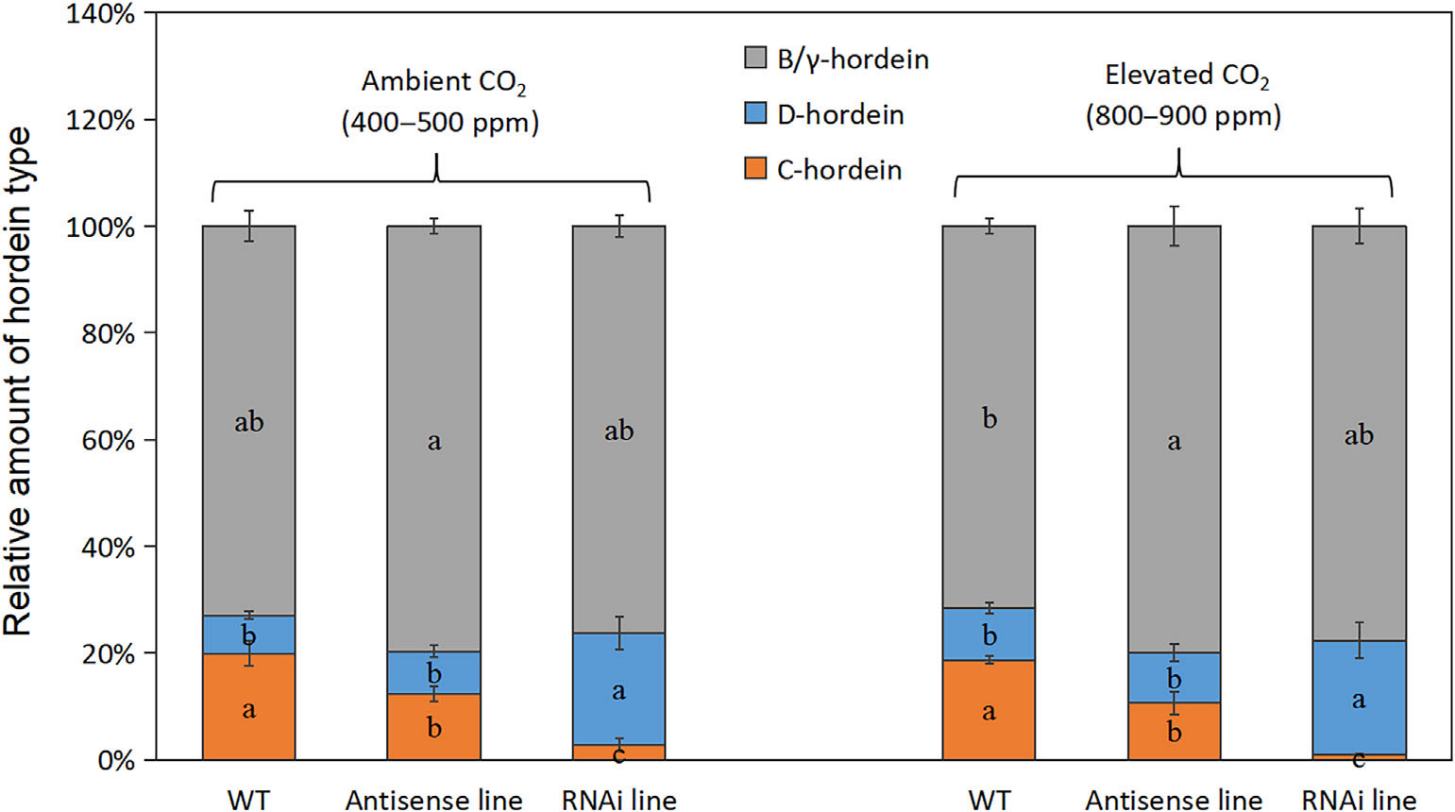
Relative amounts (%) of C‐hordein, B/γ‐hordein, and D‐hordein in grains from wild‐type plants, C‐hordein‐antisense line, and C‐hordein‐RNAi line growing under ambient or elevated atmospheric CO_2_ in greenhouse cells 2015/2016 (Experiment II). Values are means ± se (standard error) (*n* = 6). Different letters in each bar indicate significant differences (*P* <0.05, Fischer LSD) between the C‐hordein‐suppressed lines and the wild‐type for each hordein fraction

The total hordein content per unit grain dry matter was similar for the wild‐type and the C‐hordein‐suppressed lines (Table 1). Under ambient CO_2_, hordeins constituted around 40% of total grain protein in the C‐hordein‐suppressed lines and approximately 50% in the wild‐type (Table 1). However, under elevated CO_2_, the proportion of hordeins increased to around 55% of the total grain protein in the two C‐hordein‐suppressed lines, thus attaining a higher fraction compared with that in the wild‐type, where hordeins constituted 45% of total grain proteins (Table 1). SDS‐PAGE and N analyses showed that the amount of C‐hordein protein was reduced by approximately 90% in the RNAi line and 45% in the antisense line (Figures 1 and S1; Table S1). The reduction in C‐hordein was accompanied by an almost three‐fold increase in D‐hordein content in the C‐hordein‐RNAi line (Figure 1; Table S1), while the content of B/γ–hordeins was about 30% higher in both C‐hordein‐suppressed lines under elevated CO_2_ (Figure 1; Table S1).

**TABLE 1:** Hordein content in barley grains from wild‐type and hordein‐C suppressed lines grown under ambient or elevated atmospheric CO_2_

Treatment	Genotype	Hordein content	
		% of grain DM	% of total grain protein
Ambient CO_2_ 400–500 ppm	Wild‐type	4.89 ± 0.51	50.2 ± 4.8
	Antisense line	4.49 ± 0.46	42.6 ± 4.6
	RNAi lactine	4.21 ± 0.31	41.0 ± 1.8
Elevated CO_2_ 800–900 ppm	Wild‐type	4.24 ± 0.49	45.7 ± 4.9
	Antisense line	4.33 ± 0.33	55.6 ± 5.3
	RNAi line	4.27 ± 0.41	55.2 ± 5.0
*P* value for:	Barley line	0.487	0.050
	CO_2_ level	0.749	0.963
	Line × CO_2_	0.700	0.089

*Note*: Values are means ± SE (*n* = 6).

**FIGURE 2:**
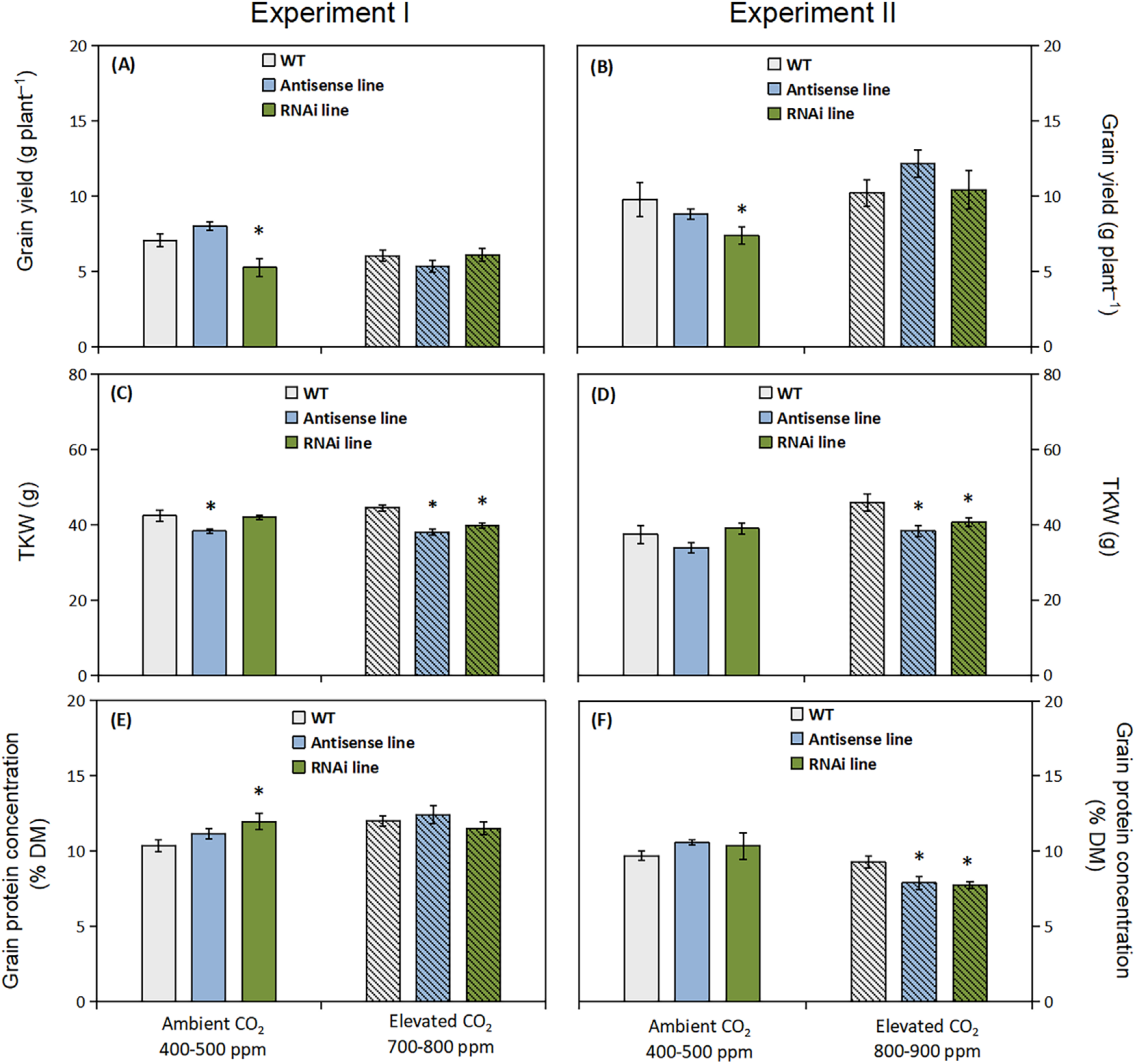
Characterization of wild‐type, C‐hordein‐antisense line, and C‐hordein‐RNAi line under ambient (open bars) or elevated atmospheric CO_2_ (hatched bars). (A) Grain yield, (C) thousand kernel weight (TKW), and (E) grain protein concentration of the wild‐type plants and C‐hordein‐suppressed lines grown in greenhouse cells 2014/2015 (Experiment I) under ambient (400–500 ppm) or elevated (700–800 ppm) atmospheric CO_2_ (*n* = 10). (B), (D), and (F) show results for the same parameters of barley plants grown in greenhouse cells 2015/2016 (Experiment II) under ambient (400–500 ppm) or elevated (800–900 ppm) atmospheric CO_2_ (*n* = 6). DM indicates dry matter. Data are presented as mean values ± se. Symbol * indicates significant differences (*P* <0.05, Fischer LSD) between the C‐hordein‐suppressed lines and the wild‐type

The effects of elevated atmospheric CO_2_ on grain yield per plant and thousand kernel weight (TKW) of the C‐hordein‐suppressed and wild‐type plants were evaluated in two independent greenhouse experiments. Plants were grown to maturity under two levels of atmospheric CO_2_ (ambient versus 700–800 ppm in Experiment I and 800–900 ppm in Experiment II). Overall, elevated CO_2_ significantly increased the grain yield in Experiment II (*P* <0.001), but not in Experiment I (Figure 2A,B), while straw yields were significantly increased (*P* <0.01) by CO_2_ in both experiments (data not shown). Under elevated CO_2_ concentration, there were no significant differences in grain yield between the three genotypes (Figure 2A,B). Growing under ambient CO_2_, the C‐hordein‐RNAi line had a significantly lower grain yield relative to the wild‐type (Figure 2A,B), due to decreased grain number per spike (data not shown). Under elevated CO_2_ concentration, there were no significant differences in grain yield between the three genotypes (Figure 2A,B). Both C‐hordein‐suppressed lines had about 10% (*P* <0.05) lower TKW compared to the wild‐type when growing under elevated CO_2_ (*P* <0.05; Figure 2C,D).

The total protein concentration was measured in the mature grains of the C‐hordein‐suppressed lines and the wild‐type (Figure 2E,F). On average, the grains contained about 10% protein, with significantly lower concentrations in the C‐hordein‐suppressed lines growing at the high level of 800–900 ppm CO_2_ in Experiment II, but not at the lower level of 700–800 ppm in Experiment I (Figure 2F). Under ambient CO_2_, there were no consistent differences in grain protein concentrations between the wild‐type and the C‐hordein‐suppressed lines in the two performed experiments (Figure 2E,F).

### 3.2 Mineral element concentrations in the whole grain and in the core endosperm

**FIGURE 3:**
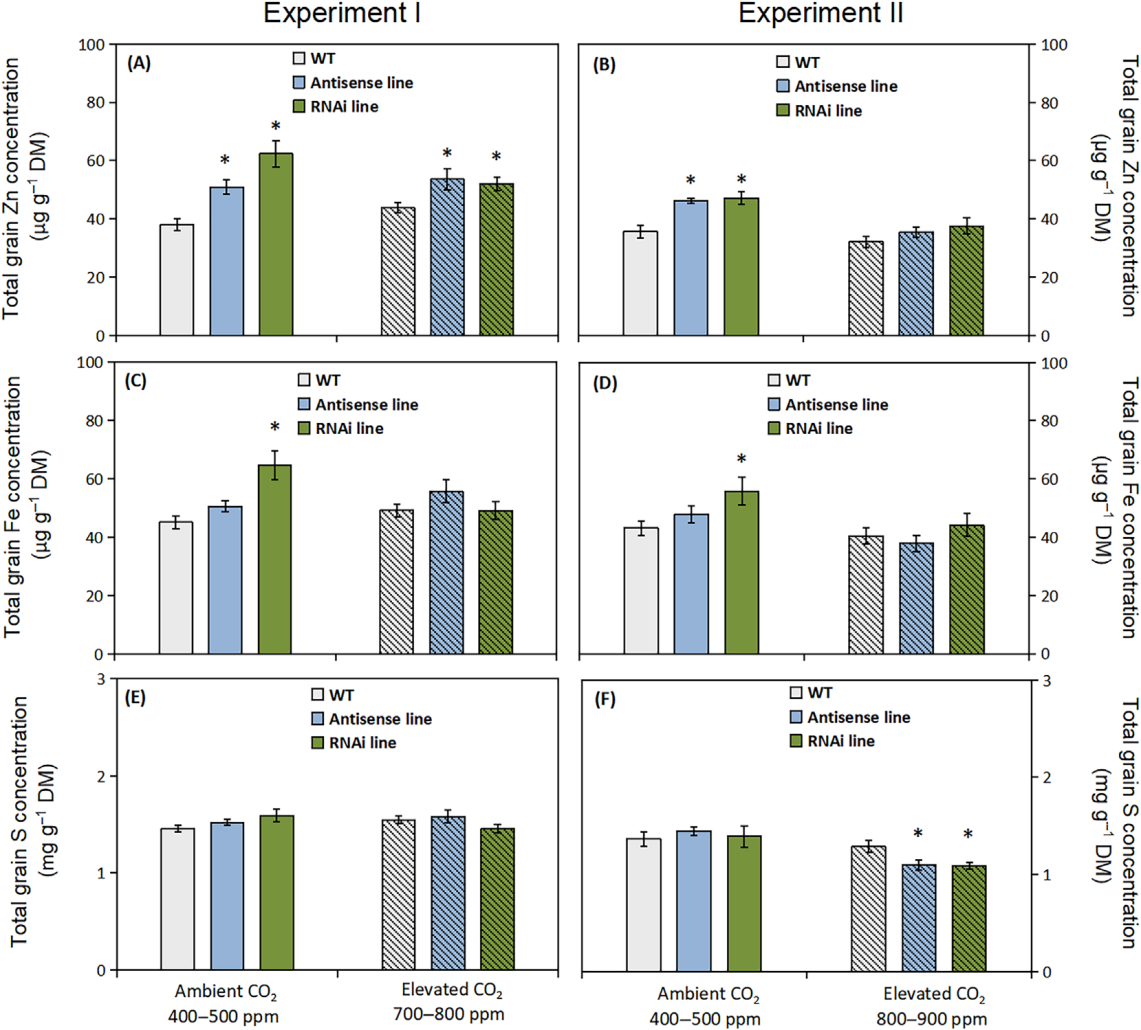
Total zinc (Zn), iron (Fe), and sulfur (S) concentrations in whole grains of wild‐type plants, C‐hordein‐antisense line, and C‐hordein‐RNAi line under ambient (open bars) or elevated atmospheric CO_2_ (hatched bars). (A) Total grain Zn, (C) total grain Fe, and (E) total grain S concentration per unit grain dry matter (DM) in C‐hordein‐suppressed and wild‐type plants grown in greenhouse cells 2014/2015 (Experiment I) under ambient (400–500 ppm) or elevated (700–800 ppm) atmospheric CO_2_ (*n* = 10). (B), (D), and (F) show results for the same parameters of barley plants grown in greenhouse cells 2015/2016 (Experiment II) under ambient (400–500 ppm) or elevated (800–900 ppm) atmospheric CO_2_ (*n* = 6). Data are presented as mean values ± se. Symbol * indicates significant differences (*P* <0.05, Fischer LSD) between the C‐hordein‐suppressed lines and the wild‐type

Mineral element concentrations in the whole grains of the C‐hordein‐suppressed lines and the wild‐type growing under ambient and elevated atmospheric CO_2_ were analyzed by ICP‐OES. Both of the two C‐hordein‐suppressed lines showed increased total grain Zn concentration, i.e. 51 and 62 μg g^−1^ dry matter for antisense line and RNAi line, respectively, compared to 38 μg g^−1^ for wild‐type plants grown under an ambient atmospheric CO_2_ in Experiment I (Figure 3A). A similar difference was observed in Experiment II where the C‐hordein‐suppressed lines both had approximately 30% higher total grain Zn concentration than the wild‐type (Figure 3B). When exposed to elevated atmospheric CO_2_ (700–800 ppm in Experiment I), the total grain Zn concentration of the two C‐hordein‐suppressed lines were still above 50 μg g^−1^ dry matter, which was 20% higher than the wild‐type (Figure 3A). In Experiment II, where the elevated atmospheric CO_2_ concentration was adjusted to 800–900 ppm, the total grain Zn concentration per unit dry matter of the C‐hordein‐suppressed lines was not significantly higher than in the wild‐type (Figure 3B).

With respect to the Fe concentration in whole grains, only the C‐hordein‐RNAi line had a significantly higher level than the wild type and only when grown under ambient atmospheric CO_2_ (Figure 3C,D). The S concentration in the whole grains of the C‐hordein‐suppressed lines showed no differences under ambient atmospheric CO_2_ (Figure 3E,F). Under elevated CO_2_, the C‐hordein‐suppressed lines had lower grain S concentration compared to the wild‐type at the high level of CO_2_ (800–900 ppm in Experiment II), but not at the low level (700–800 ppm in Experiment I; Figure 3F).

In order to further evaluate the mineral status of the core endosperm, the barley grains were polished to remove the outer layers (testa and aleurone) and the embryo. The mineral concentrations in the endosperm and the removed outer parts, as well as the total mineral concentration in whole grains before polishing, were analyzed by ICP‐OES. The recoveries for Zn or Fe were within 85–115% of the total Zn or Fe in each grain, indicating only minor losses of material and contamination during polishing and sample handling (data not shown).

**FIGURE 4:**
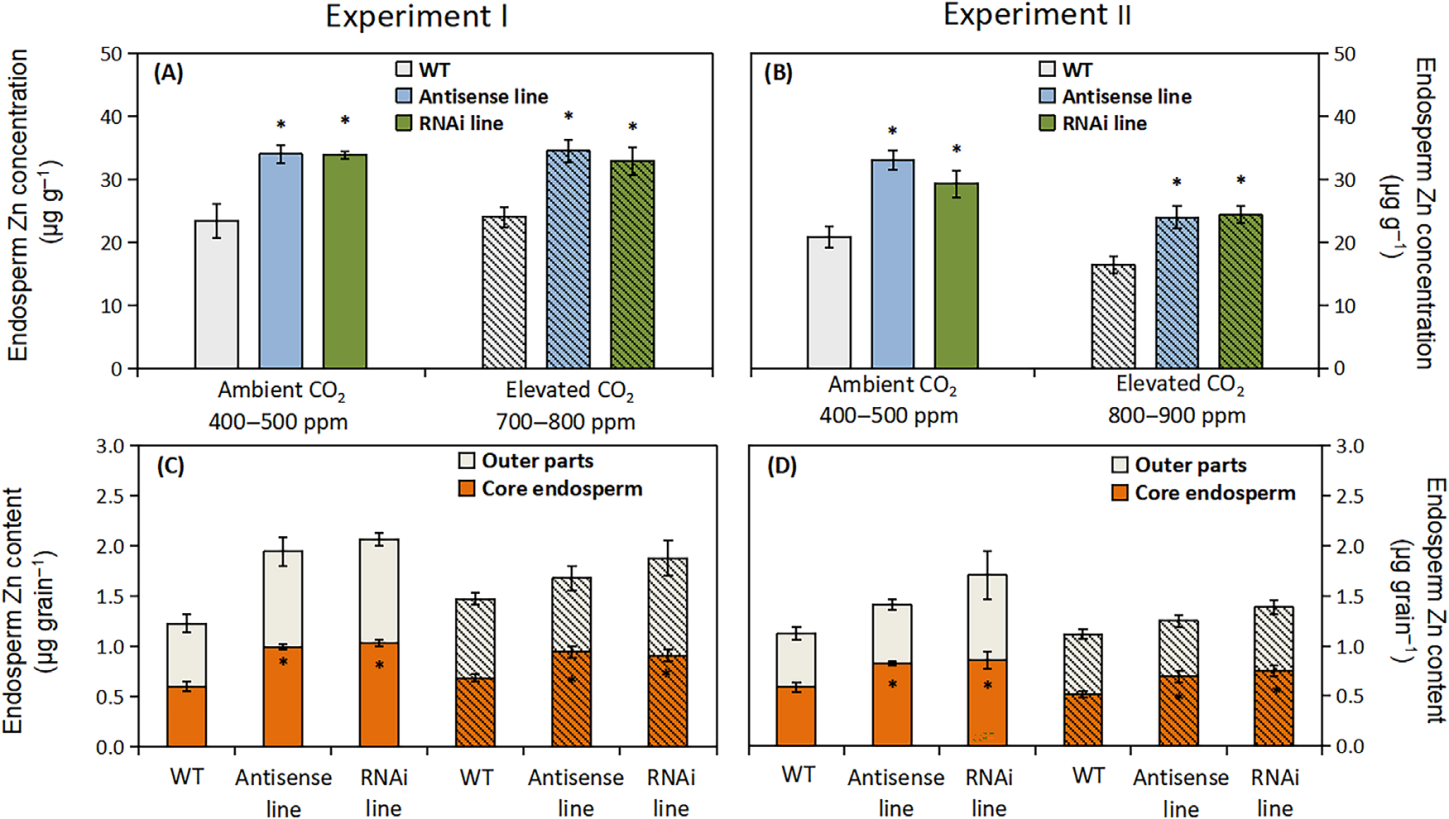
Endosperm Zn per unit endosperm dry matter and per whole grain of wild‐type plants, C‐hordein‐antisense line, and C‐hordein‐RNAi line grown under ambient (open bars) or elevated atmospheric CO_2_ (hatched bars). (A) Zn concentration in the core endosperm and (C) Zn content in the endosperm and outer grain layers (testa, aleurone, and embryo) of wild‐type and C‐hordein‐suppressed plants grown in greenhouse cells 2014/2015 (Experiment I) under ambient (400–500 ppm) or elevated (700–800 ppm) atmospheric CO_2_ (*n* = 4). (B) and (D) show results for the same parameters of grains harvested from barley plants grown in greenhouse cells 2015/2016 under ambient (400–500 ppm) or elevated (800–900 ppm) atmospheric CO_2_ (*n* = 4). Data are presented as mean values ± se. Symbol * indicates significant differences (*P* <0.05, Fischer LSD) between the C‐hordein‐suppressed lines and the wild‐type

The two C‐hordein‐suppressed lines had in all treatments significantly higher Zn (37–58%) concentration in the endosperm compared to the wild‐type (Figure 4A,B). When exposed to an elevated atmospheric CO_2_ concentration of 800–900 ppm, the endosperm Zn concentration in both hordein‐C suppressed lines decreased relative to ambient CO_2_ (Figure 4B), but the C‐hordein‐suppressed lines still had almost 50% higher endosperm Zn concentration than the wild‐type (Figure 4B). The higher Zn concentration in the core endosperm of the C‐hordein‐suppressed lines resulted in a corresponding increase in the Zn content in the core endosperm of each grain (Figure 4C,D). There were no significant differences between the C‐hordein‐suppressed lines and wild‐type with respect to the Zn content of the outer grain parts, including the testa, aleurone and embryo (Figure 4C,D).

**FIGURE 5:**
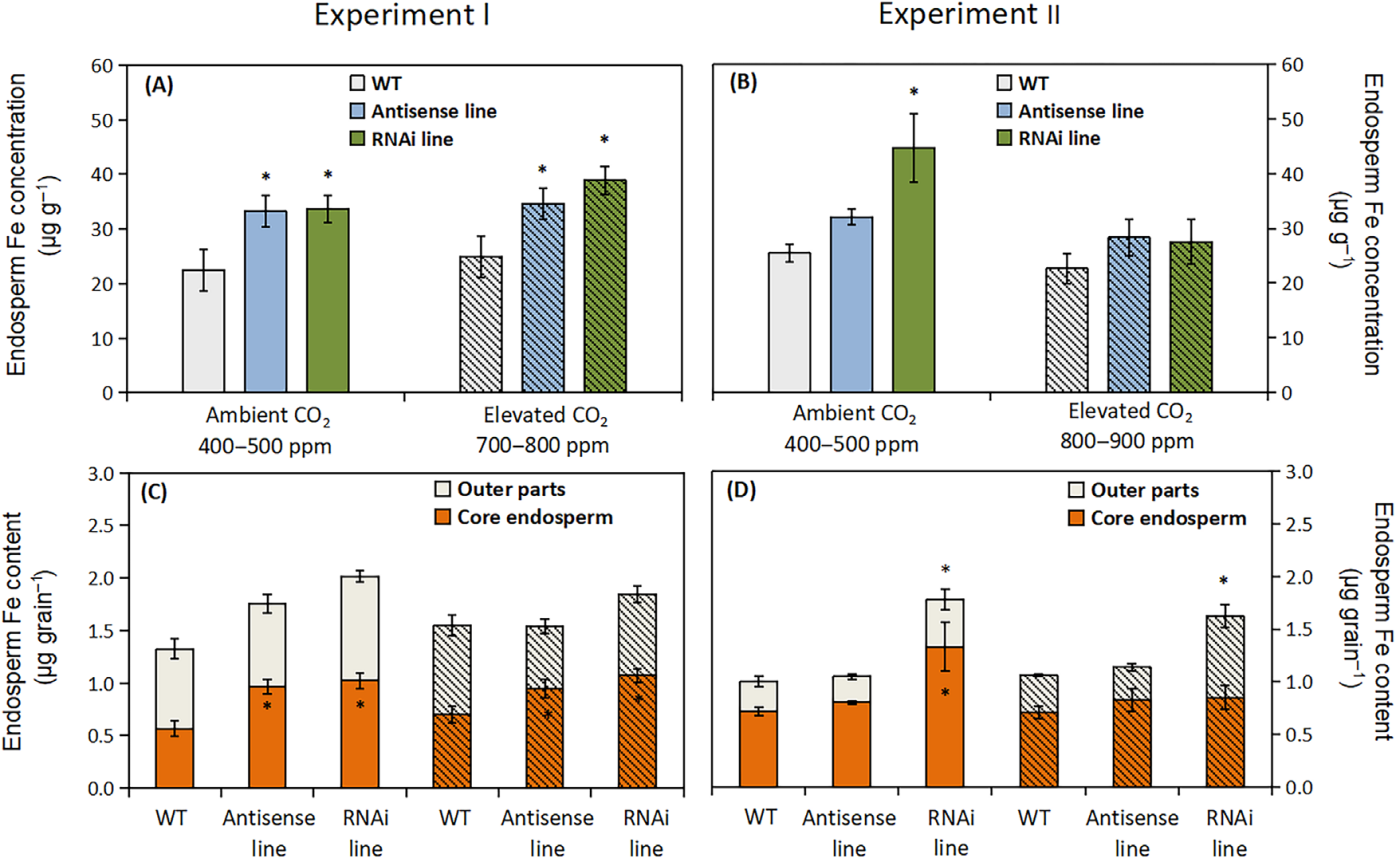
Endosperm Fe per unit endosperm dry matter and per whole grain of wild‐type plants, C‐hordein‐antisense line, and C‐hordein‐RNAi line grown under ambient (open bars) or elevated atmospheric CO_2_ (hatched bars). (A) Fe concentration in the core endosperm and (C) Fe content in the endosperm and outer grain layers (testa, aleurone, and embryo) of wild‐type and C‐hordein‐suppressed plants grown in greenhouse cells 2014/2015 (Experiment I) under ambient (400–500 ppm) or elevated (700–800 ppm) atmospheric CO_2_ (*n* = 4). (B) and (D) show results for the same parameters of grains harvested from barley plants grown in greenhouse cells 2015/2016 under ambient (400–500 ppm) or elevated (800–900 ppm) atmospheric CO_2_ (*n* = 4). Data are presented as mean values ± se. Symbol * indicates significant differences (*P* <0.05, Fischer LSD) between the C‐hordein‐suppressed lines and the wild‐type

With respect to Fe, the endosperm concentration in the C‐hordein‐suppressed lines was in Experiment I significantly higher (22–76%) than in the wild‐type at both ambient and elevated atmospheric CO_2_ (Figure 5A). In Experiment II, where the plants were growing at higher light intensity and N supply than in Experiment I, and accordingly produced higher grain yields (Figure 2B), only the RNAi line showed higher endosperm Fe concentration than the wild‐type at ambient CO_2_ (Figure 4B). None of the two C‐hordein‐suppressed lines showed higher endosperm Fe concentration than the wild‐type at elevated CO_2_ (Figure 5D), but the Fe content in the outer grain parts was higher in the RNAi line than in the two other lines (Figure 5D).

The Mn concentration in the endosperm did not differ between the C‐hordein‐suppressed lines and the wild‐type (Figure S1 A,B). The magnesium (Mg) concentration, however, tended to be higher in the C‐hordein‐suppressed lines compared to the wild‐type (Figure S1C,D), while the opposite was the case for potassium (K) under elevated CO_2_ (Figure S1E,F). Other element concentrations, including copper (Cu), calcium (Ca), and phosphorus (P), were not significantly different in the C‐hordein‐suppressed lines compared with the wild‐type (data not shown).

### 3.3 Mineral distribution in the grain

**FIGURE 6:**
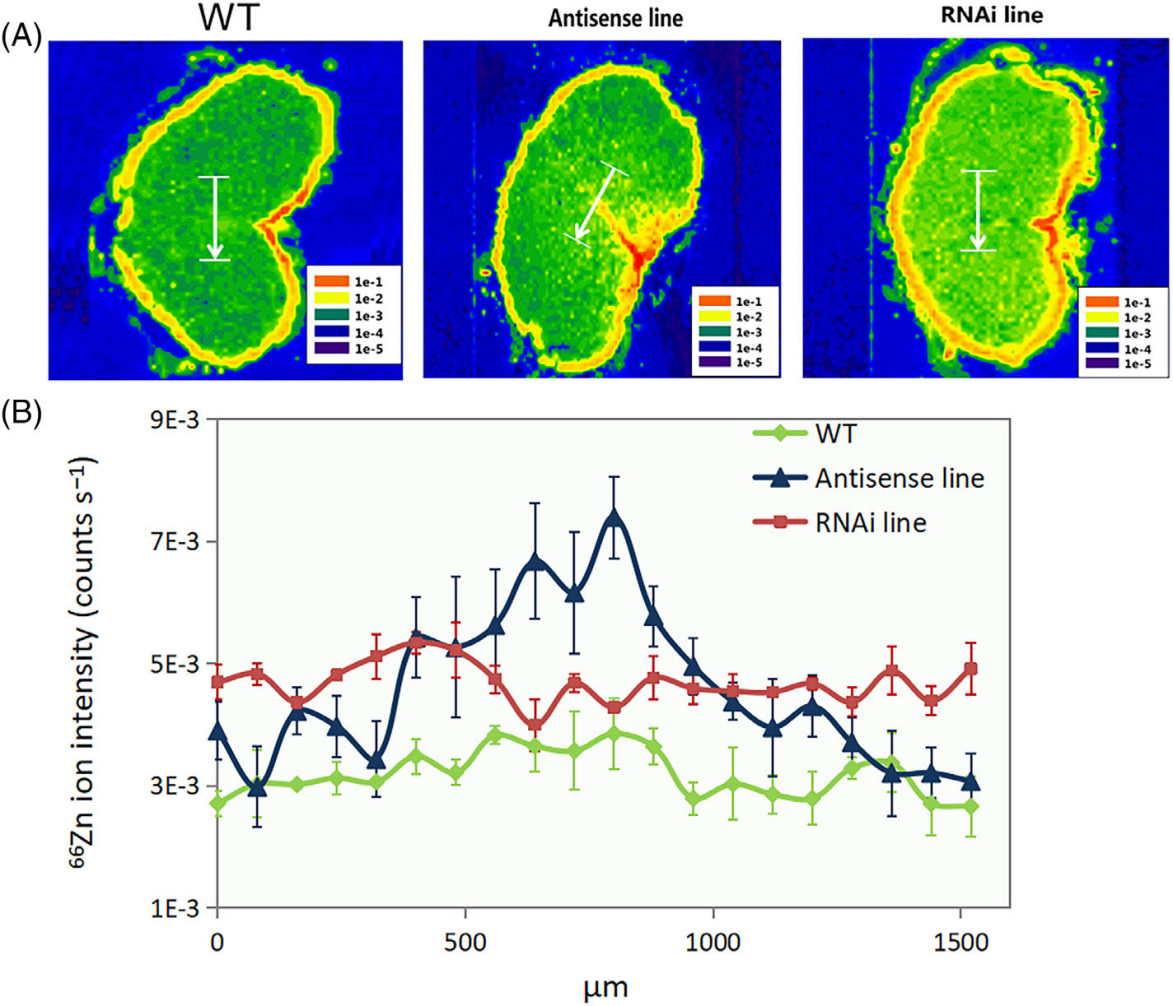
Distribution of ^66^Zn in cross‐sections of grains of wild‐type plants, C‐hordein‐antisense line, and C‐hordein‐RNAi line grown in greenhouse cells 2015/2016 (Experiment II) under ambient (400–500 ppm) atmospheric CO_2_. Element images were obtained using laser ablation‐inductively coupled plasma‐mass spectrometry (LA‐ICP‐MS). (A) Heat map for the distribution of ^66^Zn (red color represents the highest intensity and the purple color the weakest intensity). (B) ^66^Zn intensities (counts s^−1^) of the line scans across the core endosperm of the grains, corresponding to the white line in (A). Each line represents the mean of 60 line scans from three individual grains (20 lines per grain) from the area represented by the white line in (A). All data points were normalized to endogeneous ^13^C. Values are means ± se (*n* = 3)

**FIGURE 7:**
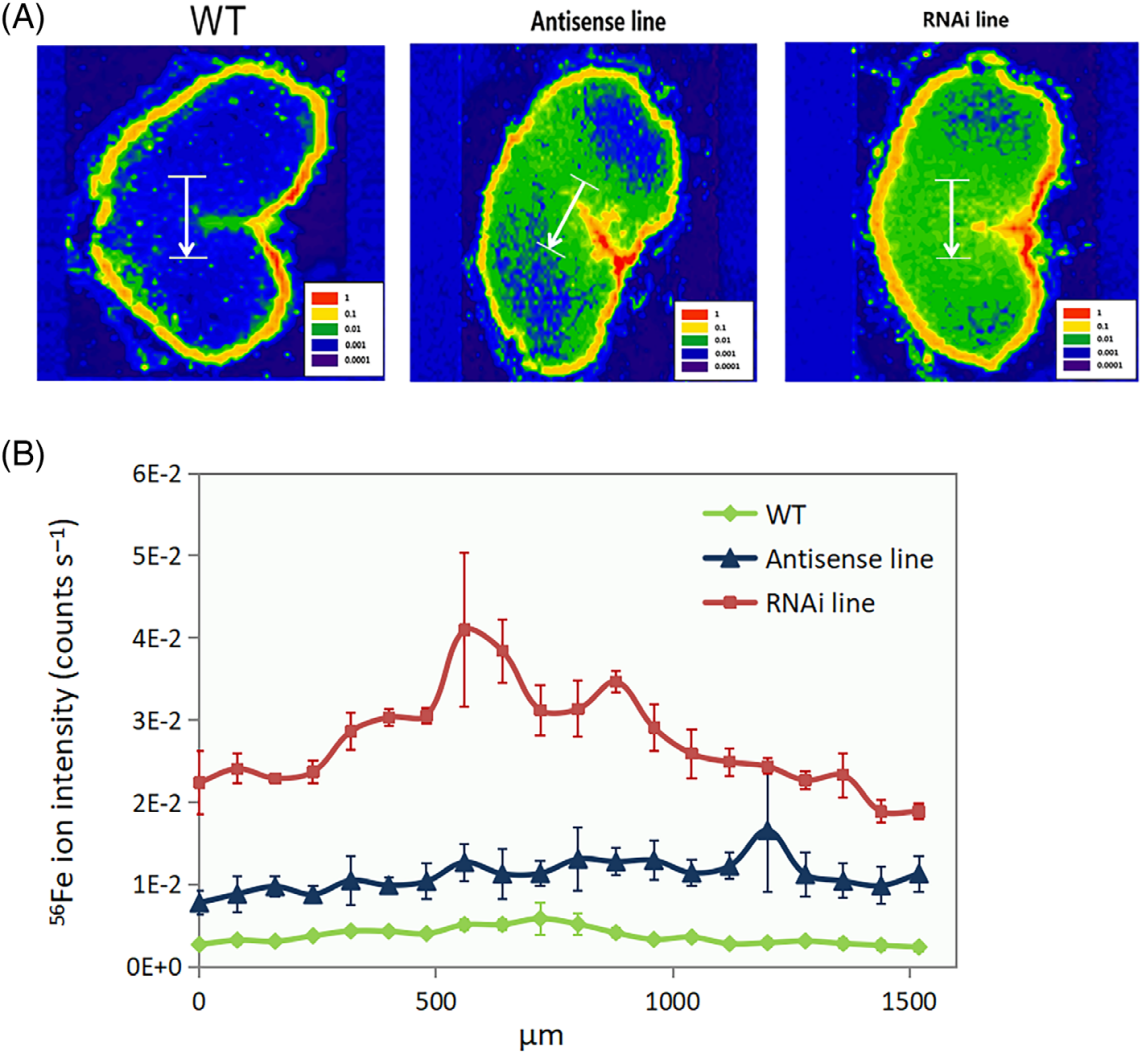
Distribution of ^56^Fe in cross‐sections of grains of wild‐type plants, C‐hordein‐antisense line, and C‐hordein‐RNAi line grown in greenhouse cells 2015/2016 (Experiment II) under ambient (400–500 ppm) atmospheric CO_2_. Element images were obtained using laser ablation—inductively coupled plasma‐mass spectrometry (LA‐ICP‐MS). (A) Heat map for the distribution of ^56^Fe (red color represents the highest intensity and the purple color the weakest intensity). (B) ^56^Fe intensities (counts s^−1^) of the line scans across the core endosperm of the grains, corresponding to the white line in (A). Each line represents the mean of 60 line scans from three individual grains (20 lines per grain) from the area represented by the white line in (A). All data points were normalized to endogeneous ^13^C. Values are means ± se (*n* = 3)

**FIGURE 8:**
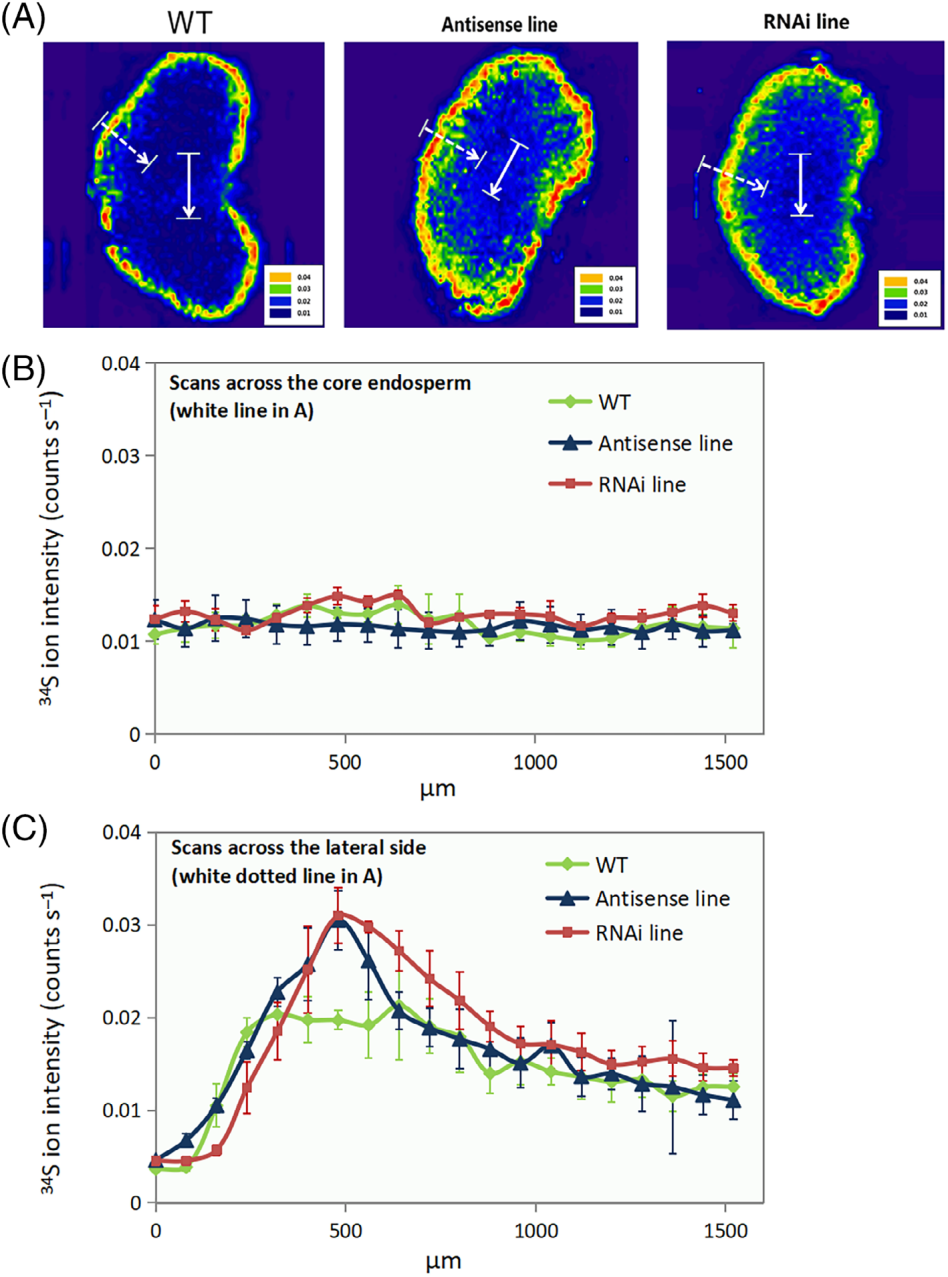
Distribution of ^34^S in cross‐sections of grains of wild‐type plants, C‐hordein‐antisense line, and C‐hordein‐RNAi line grown in greenhouse cells 2015/2016 (Experiment II) under ambient (400–500 ppm) atmospheric CO_2_. Element images were obtained using laser ablation‐inductively coupled plasma‐mass spectrometry (LA‐ICP‐MS). (A) Heat map for the distribution of ^34^S (red color represents the highest intensity and the purple color the weakest intensity). (B) ^34^S intensities (counts s^−1^) of the line scans across the core endosperm of the grains, corresponding to the white line in (A). (C) ^34^S intensities (counts s^−1^) of the line scans across the testa, the aleurone layer, and the endosperm from the lateral side of the grains, corresponding to the white dotted line in (A). Each line represents the mean of 60 line scans from three individual grains (20 lines per grain) from the area represented by the white line in (A). All data points were normalized to endogeneous ^13^C. Values are means ± se (*n* = 3)

In order to provide a detailed map of the distribution of mineral elements in the grains, multi‐element bio‐imaging analysis was conducted using LA‐ICP‐MS. For both the wild‐type and the C‐hordein‐suppressed lines, the crease region and the outer layers (testa and aleurone layer) had stronger ^66^Zn and ^56^Fe ion intensities than the endosperm (Figures 6A and 7A). In the core endosperm and the crease region, the C‐hordein‐suppressed lines showed higher ^66^Zn and ^56^Fe ion intensities compared to the wild‐type (Figures 6A and 7A). An enrichment of ^34^S intensities just inside the aleurone layer was also observed (Figure 8A).

Regions of the grain showing steep changes in the concentration of ^66^Zn, ^56^Fe, and ^34^S were further explored by acquisition of LA‐ICP‐MS line scans across 1500 μm long gradients (Figures 6B, 7B, and 8B), Overall, the ^66^Zn signal in the endosperm of C‐hordein‐RNAi line were consistently about 50% higher than that of the wild‐type (Figure 6B), while the C‐hordein‐antisense line had over 100% stronger ^66^Zn signal compared to the wild‐type in the middle of the line scan (Figure 6B), representing the area in the endosperm close to the crease region (Figure 6A). Both of the two C‐hordein‐suppressed lines also showed consistently higher ^56^Fe intensities in the core endosperm of the grains. The ^56^Fe signals were 6 and 2 times higher for the RNAi line and the antisense line, respectively, relative to the wild‐type (Figure 7B). Gradients of ^34^S were similar in the core endosperm of the C‐hordein‐suppressed lines and the wild‐type (Figure 8B). However, close to the aleurone layer, the ^34^S signal was 40%–50% higher in the C‐hordein‐suppressed lines than in the wild‐type (Figure 8C).

## 4 DISCUSSION

### 4.1 Yield responses to CO_2_ treatments

Elevated CO_2_ significantly increased the grain yield in Experiment II (*P* <0.001), but not in Experiment I (Figure 2A,B), while straw yields in both experiments were significantly increased (*P* <0.01; data not shown). In FACE experiments embracing more than 100 barley genotypes, Ingvordsen et al. (2015, 2016) observed an average grain yield increase of 17%. Similar magnitude of yield increase for barley was reported in a meta‐analysis by Taub et al. (2008). Effects of elevated CO_2_ depend on the intensity of other growth factors, in particular N and water, where limitations will reduce yield responses (Kimball, 2016). In the present experiments, plants were grown at low N supply in order to obtain realistic grain protein levels matching those in field‐grown barley plants. This resulted in grain protein concentrations around 10% (Figure 2E,F) as opposed to previously reported values of 17% for C‐hordein antisense lines grown with high N supply in greenhouses (Lange et al., 2007). The grain protein concentration in the more than 100 barley genotypes included in the FACE experiments by Ingvordsen et al. (2015, 2016) ranged between 10 and 18% with only a couple of lines representing the low N status of the plants in the present study. The limitation in N supply may have reduced the growth responses to elevated CO_2_, particularly in Experiment I, where the N supply and CO_2_ were lower than in Experiment II. Additional factors may have influenced the yield response, including light limitation and pot size. The applied light intensity of 400–500 μmol m^−2^ s^−1^ should be sufficient to analyze the effect of high CO_2_ in barley (Kromer et al., 1993). The plants were grown in relatively small pots containing 2 L soil and the volume of the rooting medium has in some cases (Bourgault et al., 2017), but not in others (Taub et al., 2008) been found to affect the response to CO_2_. Any effect of pot size must be assumed strongly confounded with nutrient supply.

### 4.2 Grain weight and protein concentration of the C‐hordein‐suppressed lines

Overall, the average grain weight (based on the TKW) of the C‐hordein‐suppressed lines (39 mg per grain; Figure 2A,B) was within the normal range, agreeing with previous observations for the C‐hordein‐suppressed lines (Lange et al., 2007; Sikdar et al., 2016). When exposed to elevated CO_2_, the TKW of the wild‐type increased while that of the C‐hordein‐suppressed lines did not respond, thus becoming 13% lower than in the wild‐type (Figure 2C,D). Variable effects of elevated atmospheric CO_2_ on TKW in wheat have been reported. Increasing TKWs were observed by Högy and Fangmeier (2008) and Fernando, Panozzo, Tausz, Norton, Neumann, et al. (2014); Fernando, Panozzo, Tausz, Norton, Fitzgerald, et al. (2014), while Kimball et al. (2001) and Högy et al. (2009) reported no change or even decreasing TKW. The effect of elevated CO_2_ on TKW seems to differ greatly among wheat cultivars (Zhang et al., 2015), and also to depend on other environmental factors such as temperature (Chaturvedi et al., 2017) and irrigation (Fernando, Panozzo, Tausz, Norton, Neumann, et al., 2014). The C‐hordein‐suppressed RNAi line studied here had an altered grain protein composition (Sikdar et al., 2016) and may thus have a different accumulation pattern of starch and proteins during grain development (Wroblewitz et al., 2014), resulting in no TKW response to elevated CO_2_. With respect to total grain protein concentration, the wild‐type plants did not show a significant decline under elevated CO_2_ (Figure 2E,F), whereas the grain protein concentration of the C‐hordein‐suppressed lines were reduced under 800–900 ppm atmospheric CO_2_ (Figure 2F). The lower grain protein concentration of the C‐hordein‐suppressed lines in response to CO_2_ enrichment may reflect reduced reallocation of N and reduced grain loading of amino acids due to the restricted C‐hordein synthesis (Postles et al., 2016; Sikdar et al., 2016), which may also lead to a reduced accumulation of carbohydrates under elevated CO_2_ (Aranjuelo et al., 2013; Wroblewitz et al., 2013).

### 4.3 Increased Zn and Fe concentration in the grain of the C‐hordein‐suppressed lines

The lower amount of C‐hordeins in the grain storage proteins resulted in increased Zn concentration not only in the whole grains but also in the core endosperm (Figures 3A,B, 4, and 6). Growing under ambient CO_2_ conditions, the Zn concentrations in the endosperm of the C‐hordein‐suppressed lines were about 50% higher than in the wild‐type (Figure 4A,B) and had thus reached the breeding target of 33 μg g^−1^ dry weight (Bouis & Welch, 2010). When exposed to an elevated CO_2_ level of 800–900 ppm, both the C‐hordein‐suppressed lines and the wild‐type showed a decline in endosperm Zn concentration relative to ambient CO_2_. This decline is in agreement with the observations made in other CO_2_ enrichment studies (Guo et al., 2015; Myers et al., 2014; Uddling et al., 2018). However, the C‐hordein‐suppressed lines still had a higher Zn level in the endosperm compared to the wild‐type under elevated CO_2_ (Figure 4B).

Proteins represent the major sink for Zn and other metals in the endosperm. Due to the low phytate content in the endosperm, this tissue has a relatively high Zn bio‐availability to humans and monogastric animals (Cakmak, Kalayci, et al., 2010; Velu et al., 2014). In durum wheat, nearly 80% of the total endosperm Zn grain was associated with water‐insoluble proteins, possibly with a large fraction of Zn bound to γ‐gliadins and high‐molecular‐weight glutenin proteins (Persson et al., 2016). In barley, a positive correlation between B‐hordein transcripts and grain Zn concentration after Zn fertilization was revealed (Uddin et al., 2014). Using Zn‐IMAC (Zn ion affinity chromatography) and Nano LC‐MS/MS (nanoscale liquid chromatography coupled to tandem mass spectrometry), Dionisio et al. (2018) identified that in the endosperm, B‐hordeins, and to a smaller extent D‐ and γ‐hordeins, were abundant Zn binding proteins. In the present study, the reduced C‐hordein content was accompanied by an increased proportion of D‐ and B/γ‐hordeins in the endosperm storage proteins (Figure 1; Table S1). Since the B/γ‐ and D‐hordeins likely bind more Zn, the increased level of these protein fractions was probably the main factor contributing to the higher endosperm Zn concentration in the C hordein‐suppressed lines.

Besides the enriched Zn concentration, the suppression of C‐hordein synthesis also resulted in an increased endosperm Fe concentration (Figure 5A; Figure 7). Growing under ambient CO_2_, both of the two C‐hordein‐suppressed lines had ~50% higher Fe concentration in the endosperm compared to the wild‐type in Experiment I (Figure 5A). In Experiment II, only the RNAi line showed higher endosperm Fe concentration than the wild‐type at ambient CO_2_ (Figure 4B), while at elevated CO_2_ the Fe content in the outer grain parts was higher in the RNAi line than in the two other lines (Figure 5D). The plant growth conditions in Experiment II differed from those in Experiment I in terms of higher light intensity and N supply. This resulted in higher grain yields, most pronouncedly under elevated CO_2_ (compare Figures 2A,B), which may have affected the loading and deposition of Fe in the endosperm.

Grain loading with Zn and Fe is determined by a range of transporters (Che et al., 2019; Connorton et al., 2017; Menguer et al., 2017) and ligands (Balk et al., 2019; Clemens, 2019). The concomitant enrichment of Zn and Fe in the endosperm of the C‐hordein suppressed lines (Figures 4 and 5) suggest that the altered protein composition affected the sink strength for the two elements. Zn and Fe have a different speciation in grain tissues where Zn seems mainly bound to S‐containing peptides, while Fe is primarily associated with phytic acid in aleurone and embryo and to a lesser extent in endosperm (Moore et al., 2012; Persson et al., 2009). The increased Fe concentration in the endosperm of the C‐hordein‐suppressed grains was not associated with higher phosphorus (P) concentration in the present study (data not shown), suggesting that chelators other than phytate were involved in the binding of Fe in the core endosperm as was also suggested by Balk et al. (2019).

The effect of C‐hordein suppression on the concentration of other mineral elements than Zn and Fe was also analyzed. There were no significant differences in the concentration of manganese (Mn), copper (Cu), calcium (Ca), and phosphorus (P) between the C‐hordein‐suppressed lines and the wild‐type. The magnesium (Mg) concentration was higher in the C‐hordein‐suppressed lines compared to the wild‐type (Figure S2 C,D), which may reflect changes in endosperm protein composition as Mg forms ion bonds with negative charges on proteins. Thus, except for the mobile element Mg, the endosperm of the C‐hordein‐suppressed grains were not co‐enriched with other essential elements than Zn and Fe.

### 4.4 Increased S intensities in the sub‐aleurone layer

The intensity of ^34^S in the C‐hordein‐suppressed mature grains was higher in the outer endosperm and in the sub‐aleurone layer (from the endosperm boundary toward the aleurone layer; Figure 8), relative to the wild‐type. The D‐hordeins accumulate in the embryo and starchy endosperm (Shewry & Halford, 2002), while γ‐hordeins are mainly present in the outer endosperm and sub‐aleurone areas (Chandra et al., 1999), and the B‐hordeins accumulate at the edge of the starchy endosperm along the endosperm boundary (Davies et al., 1993). Since these protein fractions are rich in S, the enrichment of ^34^S in the outer endosperm and sub‐aleurone layer of the C‐hordein‐suppressed grains, therefore likely reflects an increased proportion of B/γ‐hordeins in the sub‐aleurone layer. This local enrichment was probably not sufficiently pronounced to result in a significant increase of total S concentration on a whole‐grain basis (Figure 3E,F).

Having a tetrahedral coordination in proteins, Zn is most commonly associated with ligands provided by amino acids such as N from histidine, S from cysteine and O from aspartate and glutamate (Auld, 2001; McCall et al., 2000). The increased level of D‐hordeins in the starchy endosperm that are rich in cysteine may therefore be beneficial for Zn accumulation. In addition, the higher accumulation of B‐hordeins with more cysteine in the endosperm of C‐hordein‐suppressed lines at early stage of grain development may promote Zn accumulation in the endosperm (Davies et al., 1993; Ozturk et al., 2006). However, in the mature grains, the higher Zn intensities in the core endosperm (close to the crease region) of the C‐hordein‐suppressed (Figure 6) lines did not co‐localize with the ^34^S enrichment (Figure 8). The higher S‐abundance rather occurred in the outer endosperm, suggesting that hordeins accumulated at the periphery of the starchy endosperm toward the apex during the later stages of grain development (Davies et al., 1993). Thus, the S‐rich hordeins could also play a role in transfer of Zn to the central endosperm during the late grain development.

The fact that changes in the composition of storage proteins in barley grains may increase the concentration of Zn and Fe in the endosperm is relevant for efforts to develop cereals with a higher density of bioavailable micronutrients. With respect to both conventional breeding and genetic engineering, barley is an important model organism for studying the molecular mechanisms involved in the loading of Zn and Fe into the endosperm (Detterbeck et al., 2019). Limited knowledge is available on the specific binding forms of micronutrients in the endosperm (Dionisio et al., 2018; Persson et al., 2016), but as evidenced in the present work, storage protein composition is important for determining the sink strength of the endosperm and, thus, the capacity for final deposition of micronutrients. Along the same line, Wu et al. (2018) showed that overexpression of the Fe storage protein ferritin in the endosperm of rice greatly increased the Fe content by creating a sink to sequester Fe. Knowledge about specific barley genes, e.g. nicotianamine synthase (NAS) genes, have also in several cases been used for obtaining rice and wheat with higher Zn and Fe levels (Majumder et al., 2019; Masuda et al., 2009).

The impact of future climate change on the concentration of mineral elements in the edible parts of crop plants will not alone depend on elevated atmospheric CO_2_. This is the case because there will be an interaction with other climatic factors, e.g. temperature, that will change along with CO_2_ (Pilbeam, 2015). Across a collection of over 100 barley lines, elevated CO_2_ (700 ppm) resulted in a 5% decrease in grain protein, while increasing temperature (5°C) caused 29% increase in grain protein (Ingvordsen et al., 2015, 2016). However, when plants were exposed to combined elevated CO_2_ and temperature, the increase in grain protein was only 8% (Ingvordsen et al., 2015, 2016). In all cases, elevated temperature caused a substantial (approximately 30%) decrease in grain yields. The consequences for grain mineral concentrations in barley of interactions between climate factors have not yet been studied, while for other species both negative and positive effects have been reported (Soares et al., 2019).

## 5 CONCLUSIONS

We conclude that suppression of the C‐hordein content in barley grains may provide higher Zn and Fe concentrations in the core endosperm. This conclusion is based on observations of an increase in endosperm Zn concentration up to 50%, which was maintained also in plants growing under elevated atmospheric CO_2_ concentration. Under ambient CO_2_, the C‐hordein‐suppressed lines also had on average 50% higher Fe concentration in the endosperm, and around 30% higher Fe concentration during elevated CO_2_ (700–800 ppm). Hence, suppression of C‐hordeins in barley has potential to supply humans and animals with adequate Zn and Fe, today and in a future climate scenario with elevated CO_2_ levels.

## CONFLICT OF INTEREST

The authors declare that they have no conflict of interest.

## AUTHOR CONTRIBUTIONS

Yajie Gao and Jan K. Schjoerring designed the research. Yajie Gao performed plant cultivation and analyzed plant growth parameters, total nitrogen, and mineral elements. Eva Vincze contributed seeds and made the SDS‐PAGE analysis of hordein fractions. Daniel P. Persson carried out the LA‐ICP‐MS analyses. All authors contributed to data analysis, interpretation, and discussion. Yajie Gao and Jan K. Schjoerring wrote the manuscript and revised it together with Daniel P. Persson and Eva Vincze. All authors have read and approved the final manuscript.

## Supporting information


**Table S1** Content of total hordein, C‐hordein, B/γ‐hordein, and D‐hordein in barley grain from wild‐type and C‐hordein suppressed plants growing under ambient or elevated atmospheric CO_2_.

**Figure S1** The SDS‐PAGE hordein patterns of the wild‐type, C‐hordein‐antisense line, and C‐hordein‐RNAi line.

**Figure S2** Endosperm manganese (Mn), magnesium (Mg), and potassium (K) per unit grain dry matter of wild‐type and C‐hordein‐suppressed plants.

## Data Availability

The data that support the findings of this study are available from the corresponding author upon reasonable request.
