# Supplementary material for: Modification of storage proteins in the barley grain increases endosperm zinc and iron under both normal and elevated atmospheric CO2
Source: Physiol Plant. 2022 Feb 2;174(1):e13624. doi: 10.1111/ppl.13624 (PMC9303220; doi:10.1111/ppl.13624)
Supplement: Supplementary file 1 — Table S1 Content of total hordein, C‐hordein, B/γ‐hordein, and D‐hordein in barley grain from wild‐type and C‐hordein suppressed plants growing under ambient or elevated atmospheric CO2. [file PPL-174-0-s003.docx]

Table S1 Content of total hordein, C-hordein, B/γ-hordein and D-hordein in barley grain from wild-type and C-hordein suppressed plants growing under ambient or elevated atmospheric CO_2_.

| Treatment | Genotype | Hordein content (mg g^-1^ total grain protein) | | | |
| --- | --- | --- | --- | --- | --- |
|  |  | Total hordein | C-hordein | B/γ-hordein | D-hordein |
| Ambient CO_2_ (400-500 ppm) | WT | 501.6 ± 48.3^ab^ | 104.0 ± 20.2^a^ | 360.7 ± 25.6^ab^ | 36.9 ± 6.9^c^ |
|  | Antisense line | 426.0 ± 45.8^ab^ | 53.8 ± 10.4^b^ | 338.7 ± 36.1^ab^ | 33.5 ± 5.8^c^ |
|  | RNAi line | 409.8 ± 17.7^b^ | 11.3 ± 4.8^c^ | 313.3 ± 17.6^b^ | 85.2 ± 11.3^b^ |
|  | |  | | | |
| Elevated CO_2_ (800-900 ppm) | WT | 457.2 ± 48.6^ab^ | 86.0 ± 10.6^ab^ | 327.1 ± 35.9^ab^ | 44.1 ± 5.5^c^ |
|  | Antisense line | 555.7 ± 53.2^a^ | 59.0 ± 11.7^b^ | 447.0 ± 54.2^a^ | 49.8 ± 8.1^c^ |
|  | RNAi line | 552.1 ± 50.1^a^ | 5.75 ± 1.6^c^ | 431.0 ± 47.3^ab^ | 115.3 ± 17.2^a^ |

Data are presented as mean values ± SE (n=6). Different letters indicate significant differences (p<0.05, Fischer LSD) between the C-hordein-suppressed lines and the wild-type for each hordein fraction.
