# Supplementary figures and images for: Modification of storage proteins in the barley grain increases endosperm zinc and iron under both normal and elevated atmospheric CO2
Source: Physiol Plant. 2022 Feb 2;174(1):e13624. doi: 10.1111/ppl.13624 (PMC9303220; doi:10.1111/ppl.13624)

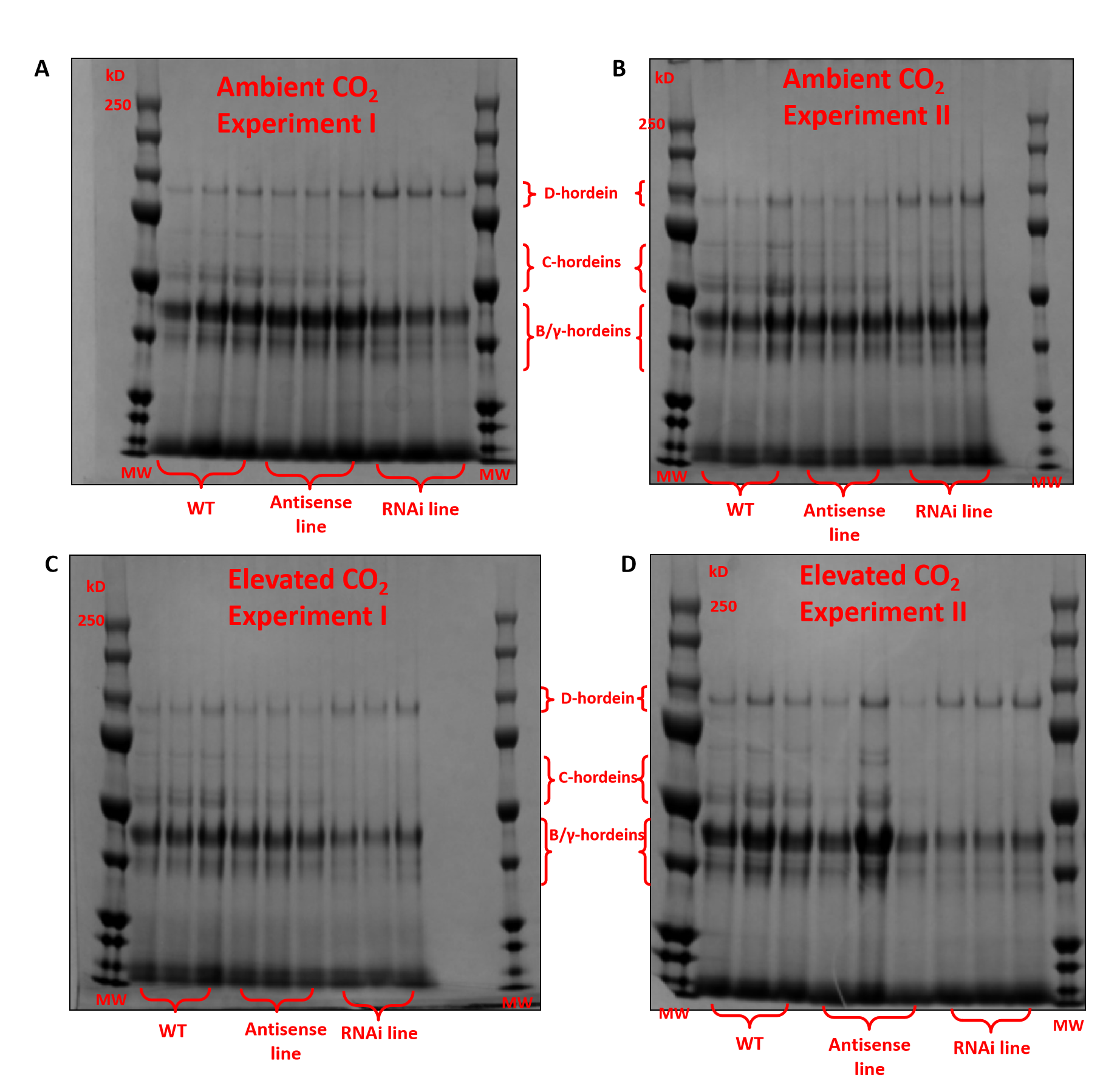

Supplement: Supplementary file 2 — Figure S1 The SDS‐PAGE hordein patterns of the wild‐type, C‐hordein‐antisense line, and C‐hordein‐RNAi line. [file PPL-174-0-s001.tif]

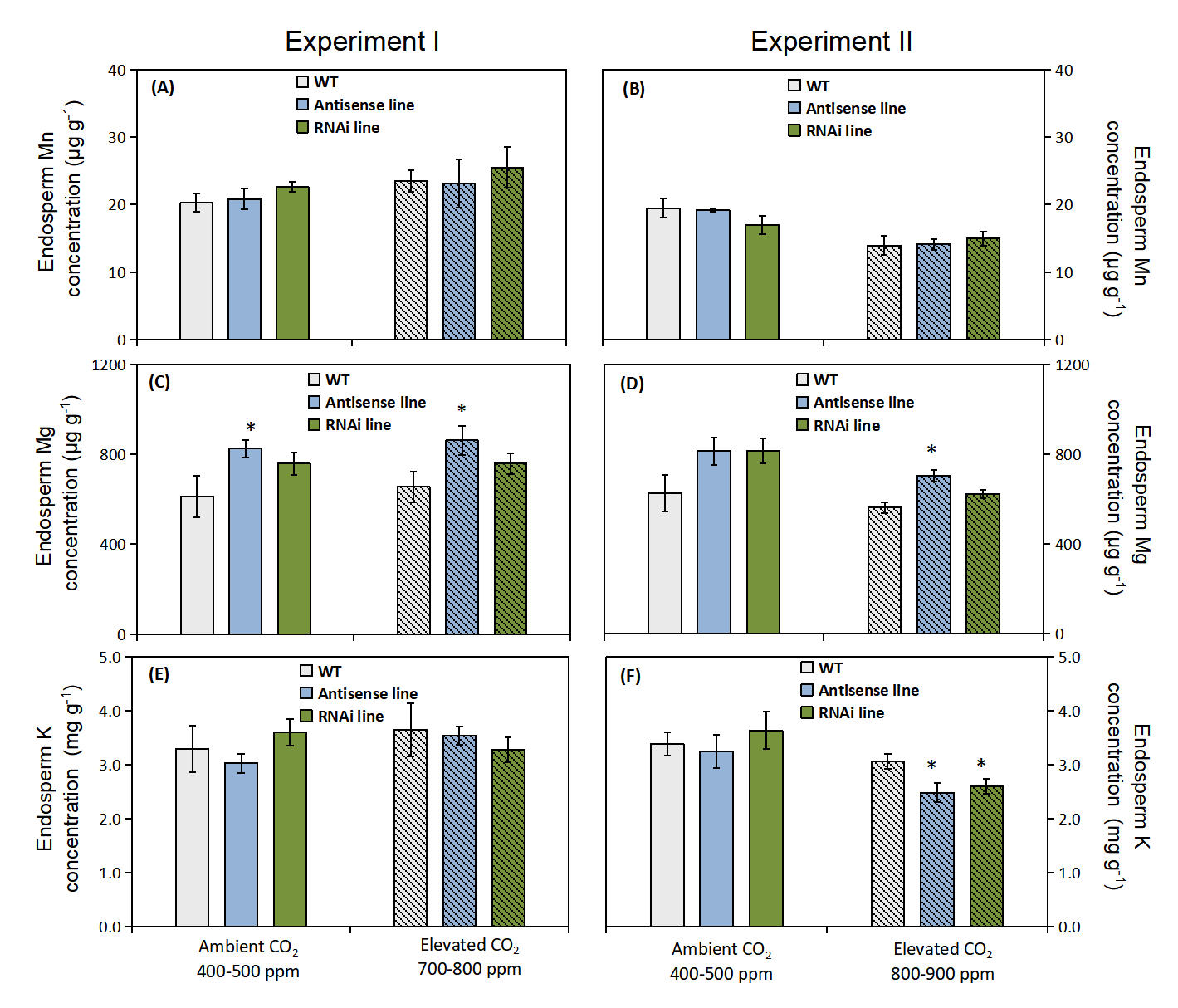

Supplement: Supplementary file 3 — Figure S2 Endosperm manganese (Mn), magnesium (Mg), and potassium (K) per unit grain dry matter of wild‐type and C‐hordein‐suppressed plants. [file PPL-174-0-s002.tif]
